# Corneal densitometry: a potential indicator for early diagnosis of Fabry disease

**DOI:** 10.1007/s00417-020-05027-6

**Published:** 2020-12-01

**Authors:** Senmao Li, Robert Siggel, Yongwei Guo, Niklas Loreck, Alexander C. Rokohl, Christine Kurschat, Ludwig M. Heindl

**Affiliations:** 1 Department of Ophthalmology, Faculty of Medicine, University of Cologne, University Hospital Cologne, Kerpener Strasse 62, 50937 Cologne, Germany; 2 Department of Ophthalmology, Helios University Hospital Wuppertal, Witten/Herdecke University, Wuppertal, Germany; 3 Eye Center, School of Medicine, Second Affiliated Hospital, Zhejiang University, Hangzhou, China; 4 Department II of Internal Medicine, Center for Molecular Medicine Cologne and Center for Rare Diseases Cologne, Faculty of Medicine, University of Cologne, University Hospital Cologne, Cologne, Germany; 5 CECAD, Faculty of Medicine, University of Cologne, University Hospital Cologne, Cologne, Germany; 6 Center for Integrated Oncology (CIO) Aachen-Bonn-Cologne-Duesseldorf, Cologne, Germany

**Keywords:** Fabry disease, Corneal densitometry, Early diagnosis, Cornea verticillata

## Abstract

**Purpose:**

To assess corneal densitometry in patients with Fabry disease (FD) and to compare corneal densitometry differences in FD patients to different corneal manifestations.

**Methods:**

Ten participants (20 eyes) with FD and 10 age-matched healthy volunteers (20 eyes) were recruited. All participants were assessed by standardized ophthalmic examinations and the corneal densitometry analysis by Pentacam HR. Densitometry measurements were analyzed in standardized grayscale units.

**Results:**

Seven patients developed conjunctival vessel tortuosity, cornea verticillata appeared in 6 patients, and two patients had Fabry cataract. Retinal vessel tortuosity occurred in 4 patients, and dilation of retinal vessels appeared in 3 patients, all symptoms occurred in both eyes. The first diagnosis of FD up to examination was 4.7 ± 3.23 years, and first ERT up to examination was 2.6 ± 2.27 years. The initial time to diagnosis was negatively related to the corneal densitometry value of the 0–2-mm (*r* = − 0.556, *p* = 0.011) and 2–6-mm (*r* = − 0.482, *p* = 0.032) zones in the posterior layer. FD group have significantly higher corneal densitometry in anterior 0–2-mm zone and 2–10-mm zone anterior and posterior layer than the control group (*p* ≤ 0.035, respectively). When divided into two groups by the existence of cornea verticillata, there was a statistically significant difference in the anterior layer, 6–10-mm zone (*p* = 0.031); in the central layer, 0–2 mm (*p* = 0.012), 2–6 mm (*p* = 0.001), 6–10 mm (*p* = 0.002), and total (*p* = 0.002); and in the posterior layer, 6–10 mm (*p* = 0.004) and total (*p* = 0.002).

**Conclusions:**

FD patients show higher corneal densitometry, and corneal densitometry may have potential for early diagnosis and reminding progress of FD.

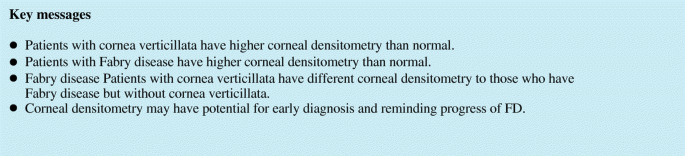



## Introduction

Fabry disease (FD) is a rare X-linked genetic disease that manifests as a lysosomal storage disorder involving glycosphingolipid metabolism abnormity due to deficient α-galactosidase A activity [1, 2]. The estimated incidence of FD in males is 1/40,000 to 1/117,000, and heterozygotic females also develop disease-related complications [3, 4]. FD has a variable presentation and involves a large number of tissues and organs, such as the kidneys, the nervous system, skin, eyes, vascular endothelium, and heart [5]. Treatment of Fabry disease focuses on supplementing missing or insufficient enzymes (α-galactosidase A) as well as stabilizing a misfolded enzyme (chaperone therapy). Enzyme replacement therapy (ERT) and chaperone therapy have widespread therapeutic efficacy in FD [6, 7].

In ocular structures, progressive deposition of glycosphingolipids due to FD causes abnormalities in the cornea, lens, and vessels of the conjunctiva and retina [8–14]. First, up to 90% of male patients with Fabry disease have cornea verticillata [15]. Second, a spoke-like lens opacity presents at the posterior capsule level known as Fabry cataract [16]. Last but not least, conjunctival and retinal vessels curve and may form aneurysms [12]. Despite these abnormities, patients’ visual acuity usually remains unaffected [17]. The eye, as an external organ, can be examined easily and non-invasively. Therefore, ocular signs may provide diagnostic or prognostic evidence of FD. Furthermore, ocular symptoms may also offer more understanding of disease prognosis and evaluation of therapies [11, 12].

Recently, corneal densitometry is feasible as a result of the developments of Scheimpflug topography systems that provide new ideas for diagnosing corneal lesions. However, few studies have focused its application on the evaluation of corneal abnormalities of FD. The purposes of this study were to assess corneal densitometry in patients with FD and to compare corneal densitometry differences in FD patients to different corneal manifestations.

## Patients and methods

In this cross-sectional study, informed consent was obtained from each subject after approval of the ethics committee of Cologne University Hospital. This observational study adhered to the Declaration of Helsinki.

### Patients

We included patients with FD who presented themselves to Cologne University Hospital during 2017.1 to 2018.5. We excluded all patients who had any history of corneal disease, corneal trauma, or eye surgery (except cataract surgery > 6 months before examination).

Medical histories, including years after the initial diagnosis of FD (less than 1 year was counted as 1 year), ophthalmic signs and symptoms, and years after the first ERT (less than 1 year was counted as 1 year), were documented. All patients underwent standardized ophthalmic examinations, with a particular focus on the cornea, lens, and conjunctival and retinal vasculatures. Ocular abnormalities were diagnosed by an experienced ophthalmologist, reporting the presence or absence of specific ocular signs. Based on these patients, we recruited 10 age-matched healthy cornea volunteers as the control group.

### Pentacam

A single expert examiner captured all Pentacam images. All measurements were taken under standardized dim-light conditions. Corneal densitometry was performed by the Pentacam HR device (Oculus, Wetzlar, Germany) over a 12-mm diameter of the cornea. Twenty-five images (1003 × 520 pixels) over different meridians of the cornea were taken, and the corneal area was divided into four concentric zones. The first zone consisted of a circular area with a 2-mm diameter at the center of the cornea; the second zone was an annular area with a diameter of 2–6 mm, surrounding the first zone; the third zone with a diameter of 6–10 mm around the second zone; and the fourth zone with a diameter of 10–12 mm surrounding the third zone.

Furthermore, densitometric values were yielded at three different depth levels of the cornea as follows: the anterior layer (120-μm thick, i.e., the superficial region of the cornea), the posterior layer (60-μm thick, i.e., the innermost part of the cornea), and the central corneal layer located between both of the layers above mentioned. The corneal densitometric values were expressed in standardized grayscale units (GSU) and as the pixel luminance per unit volume in Scheimpflug images. The measurements ranged from 0 (maximum transparency) to 100 (completely opaque cornea), according to the degree of backscattering light from the cornea.

### Statistical analysis

All data were managed using Excel 2016 for Windows (Microsoft, Redmond, WA, USA). Statistical analyses were performed with IBM SPSS Statistics 23 for Windows (IBM Corporation, Somers, NY, USA). The normality of the data distribution was tested using the Kolmogorov-Smirnov test, and the data did not fit a normal distribution. Therefore, the subgroup analysis was compared using the Mann-Whitney *U* test, and correlation was analyzed using Spearman’s correlation coefficient. We reported all data as mean and standard deviation. The level of statistical significance was set at *p* < 0.05.

## Results

### Patient demographics

This study included 10 participants (20 eyes) as FD group and 10 participants (20 eyes) age-matched healthy cornea volunteers as the control group. The mean age was 49.2 ± 15.92 years old (range, 29–71 years old). General conditions and genetic mutations for FD patients are summarized in Table 1.

**Table 1:** General conditions and genetic mutations for FD patients

No.	Sex	Age (years)	First diagnosis time (years)	First ERT time (years)	DNA	Mutation
1	F	60	6	6	c.560T>G	p.M187R
2	F	32	6	1	c.560T>G	p.M187R
3	M	53	3	2	c.132G>C	p.W44C
4	M	30	6	5	c.124A>G	p.M42V
5	M	29	2	2	c.1024C>T	p.R242*Exon7
6	F	58	1	0	c.1069C>T	p.Gln357X
7	F	37	3	2	c.376A>G	p.S126G
8	F	68	2	0	c.376A>G	p.S126G
9	M	54	6	6	c.427G>A	p.A143T
10	F	71	12	2	IVS0-10C>T IVS4-16A>G IVS6-22C>T	N/A

*N/A* unknown

In FD group, the best-corrected visual acuity (BCVA) was 0 logMAR in 8 patients (16 eyes); 0.2 logMAR in one patient (2 eyes) due to amblyopia; and 0.1 logMAR for the right eye and 0.2 logMAR for the left eye in one patient due to endocrine orbitopathy and retinal detachment. Compared with the control group, there is no statistical difference (*p* = 0.088) in visual acuity. General eye conditions for FD patients are summarized in Table 2.

**Table 2:** General eye condition of FD patients

No.	OD							OS						
	BCVA (logMAR)	IOP (mmHg)	Conjunctival vessels tortuosity	Cornea verticillata	Fabry cataract	Retinal vessels tortuosity	Retinal vessels dilation	BCVA (logMAR)	IOP (mmHg)	Conjunctival vessels tortuosity	Cornea verticillata	Fabry cataract	Retinal vessels tortuosity	Retinal vessels dilation
1	0.2	22	Y	Y	N	N	N	0.2	20	Y	Y	N	N	N
2	0	14	N	Y	N	N	N	0	12	N	Y	N	N	N
3	0	15	Y	Y	N	Y	Y	0	18	Y	Y	N	Y	Y
4	0	9	Y	Y	N	Y	Y	0	12	Y	Y	N	Y	Y
5	0	14	Y	Y	Y	Y	N	0	13	Y	Y	Y	Y	N
6	0	16	N	Y	Y	N	N	0	15	N	Y	Y	N	N
7	0	11	N	N	N	N	N	0	14	N	N	N	N	N
8	0	13	Y	N	N	Y	Y	0	11	Y	N	N	Y	Y
9	0	10	Y	N	N	N	N	0	13	Y	N	N	N	N
10	0.1	10	Y	N	N/A	N	N	0.2	4	Y	N	N/A	N	N

*F* female, *M* male, *Y* yes, *N* no, *N/A* unknown

FD patients had a mean IOP of 13.38 ± 3.69 mmHg. Of ten patients (20eyes), two (4 eyes) had myopia, one (2 eyes) had glaucoma, one (1 eye) retinal arterial occlusion in the left eye, and one (1 eye) had Graves’ ophthalmopathy and retinal detachment in the right eye. Seven (14 eyes) developed conjunctival vessels tortuosity, six (12 eyes) cornea verticillata, two (4 eyes) Fabry cataract, one (2 eyes) remained unknown lens conditions due to previous IOL implantation, four (8 eyes) retinal vessels tortuosity, and three (6 eyes) dilation of retinal vessels.

The first diagnosis of FD up to examination was 4.7 ± 3.23 (range 1 to 12) years, and the first ERT up to examination was 2.6 ± 2.27 (range 0 to 6) years. It is worth noting that two patients did not accept ERT.

### Corneal densitometry

The anterior layer had the highest corneal densitometric values, and the posterior layer had the lowest ones (*p* < 0.001).

In the anterior layer of FD group and control group, the mean corneal densitometry values were 31.5 ± 8.57 GSU and 26.95 ± 3.66 GSU in total (*p* = 0.049); 27.47 ± 3.32 GSU and 25.90 ± 2.18 GSU in the 0–2-mm zone; 26.24 ± 4.60 GSU and 23.09 ± 1.64 GSU in the 2–6-mm zone (*p* = 0.004); 34.02 ± 15.45 GSU and 25.78 ± 5.12 GSU in the 6–10-mm zone (*p* = 0.03); and 43.26 ± 15.46 GSU and 38.93 ± 12.27 GSU in the 10–12-mm zone. Statistical differences exist in the 2–6-mm, 6–10-mm, and total zones comparing with the control group.

In the central layer of FD group and control group, the mean corneal densitometry values were 21.22 ± 7.26 GSU and 18.61 ± 2.70 GSU in total; 16.17 ± 1.45 GSU and 16.66 ± 2.13 GSU in the 0–2-mm zone; 16.15 ± 4.01 GSU and 15.11 ± 1.72 GSU in the 2–6-mm zone; 25.08 ± 13.11 GSU and 18.89 ± 3.93 GSU in the 6–10-mm zone; and 30.16 ± 9.98 GSU and 27.06 ± 6.25 GSU in the 10–12-mm zone. The difference is not significant comparing with the control group.

In the posterior layer of FD group and control group, the mean corneal densitometry values were 15.94 ± 4.93 GSU and 13.12 ± 2.02 GSU in total; 11.19 ± 1.41 GSU and 9.91 ± 1.77 GSU in the 0–2-mm zone (*p* = 0.028); 11.42 ± 2.51 GSU and 9.775 ± 1.50 GSU in the 2–6-mm zone (*p* = 0.035); 18.82 ± 8.50 GSU and 13.95 ± 2.81 GSU in the 6–10-mm zone (*p* = 0.028); and 25.56 ± 9.56 GSU and 21.29 ± 3.91 GSU in the 10–12-mm zone. Statistical differences were found in every zone but the 10–12-mm zone, comparing with the control group. The results of corneal densitometry in three different depth levels of the Fabry group and the control group are summarized in Table 3.

**Table 3:** Corneal densitometric values in three different depth levels

	FD group	Control group	*p*
The anterior layer			
0–2 mm	27.47 ± 3.32	25.90 ± 2.18	0.127
2–6 mm	26.24 ± 4.60	23.09 ± 1.64	0.004**
6–10 mm	34.02 ± 15.45	25.78 ± 5.12	0.03*
10–12 mm	43.26 ± 15.46	38.12 ± 9.93	0.738
Total	31.5 ± 8.57	26.95 ± 3.66	0.049*
The central layer			
0–2 mm	16.17 ± 1.45	16.66 ± 2.13	0.341
2–6 mm	16.15 ± 4.01	15.11 ± 1.72	0.841
6–10 mm	25.08 ± 13.11	18.89 ± 3.93	0.134
10–12 mm	30.16 ± 9.98	27.06 ± 6.25	0.758
Total	21.22 ± 7.26	18.61 ± 2.70	0.355
The posterior layer			
0–2 mm	11.19 ± 1.41	9.91 ± 1.77	0.028*
2–6 mm	11.42 ± 2.51	9.775 ± 1.50	0.035*
6–10 mm	18.82 ± 8.50	13.95 ± 2.81	0.028*
10–12 mm	25.56 ± 9.56	21.29 ± 3.91	0.383
Total	15.94 ± 4.93	13.12 ± 2.02	0.056

Mean ± SD (GSU)

*p < 0.05

**p < 0.01

The initial diagnosis time was positively related to initial ERT time (*r* = 0.601, *p* = 0.005). The initial diagnosis time was negative related to the corneal densitometry value of the 0–2-mm (*r* = − 0.556, *p* = 0.011) and 2–6-mm (*r* = − 0.482, *p* = 0.032) zones in the posterior layer. However, initial ERT time was not related to the corneal densitometry value of each area. After removing two untreated cases, there was still no correlation between initial ERT time and corneal densitometry value.

### Subgroup analysis

All the eyes of FD patients were divided into no cornea verticillata group (*n* = 8) and cornea verticillata group (*n* = 12) based on the results of the slit-lamp examination. Statistical differences of corneal densitometric values between both groups are shown in Table 4.

**Table 4:** Statistical differences of corneal densitometric values between no cornea verticillata group and cornea verticillata group

	0–2 mm	2–6 mm	6–10 mm	Total
The anterior layer	0.570	0.624	0.031^*^	0.343
The central layer	0.012^*^	0.001^**^	0.002^**^	0.002^**^
The posterior layer	0.734	0.135	0.004^**^	0.002^**^

*p < 0.05

**p < 0.01

As for the anterior layer, a statistically significant difference was found in the 6–10-mm zone (*p* = 0.031) between patients with and without cornea verticillata. Concerning the central layer, the total zone, and each zone presented statistically significant differences between both groups with a *p* value of 0.012, 0.001, 0.002, and 0.002 in the 0–2-mm, 2–6-mm, 6–10-mm, and total zones, respectively. Regarding the posterior layer, significant differences were indicated in the 6–10-mm zone (*p* = 0.004) and the total zone (*p* = 0.002).

**Fig. 1:**
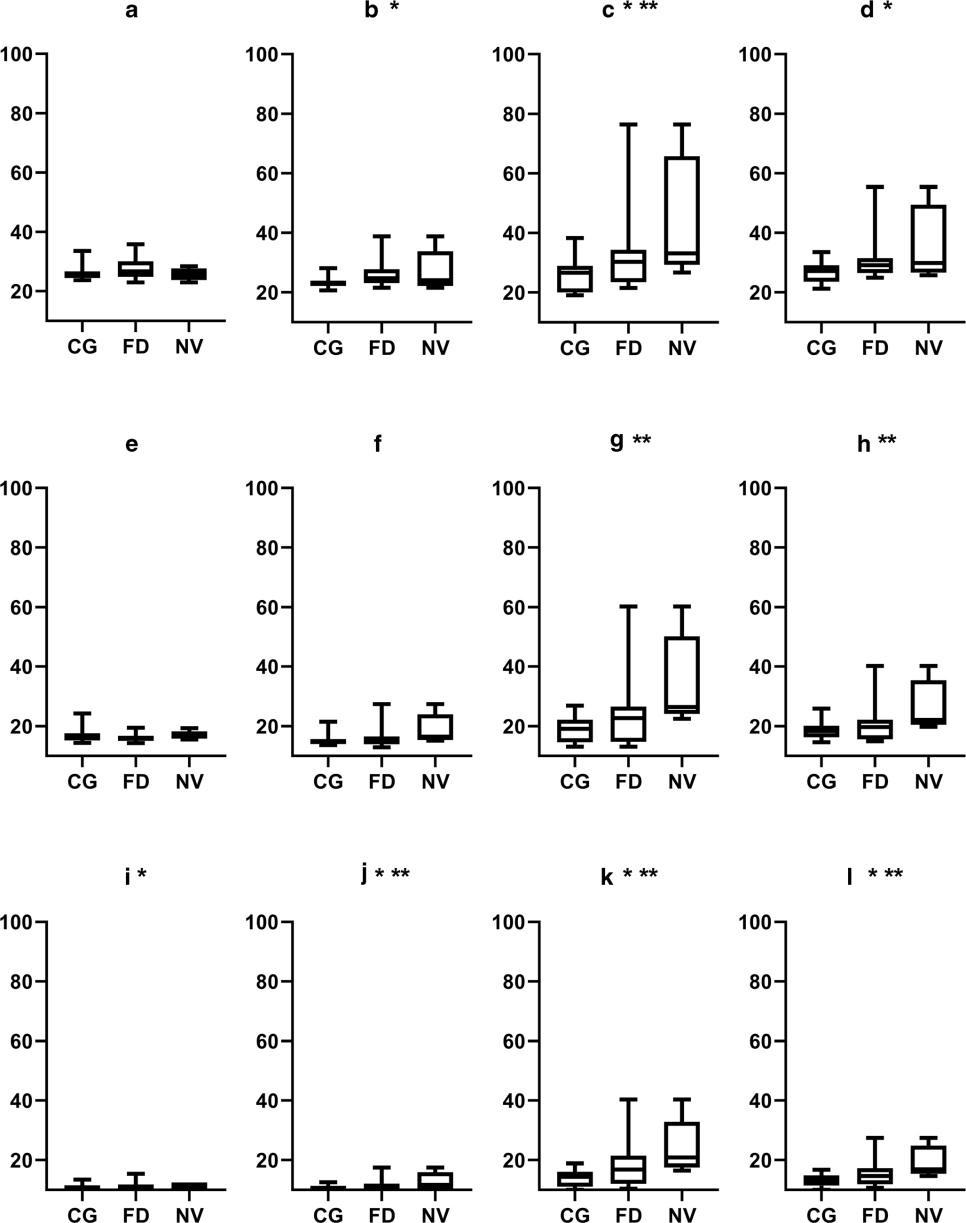
Densitometric values in FD and control individuals with and without cornea varicellate. *Y*-axis is densitometric values (GSU). CG control group, FD Fabry disease group, NV FD without cornea verticillata group. **a**, **b**, **c**, and **d** belong to the anterior layer; **e**, **f**, **g**, and **h** belong to the central layer; **i**, **j**, **k**, and **l** belong to the posterior layer. **a**, **e**, and **i** are 0–2-mm zones; **b**, **f**, and **j** are 2–6-mm zones; **c**, **g**, and **k** are 6–10-mm zones; **d**, **h**, and **l** are total layers. *Statistical differences occur between CG and FD. **Statistical differences occur between CG and NV

However, there were no statistical differences between the cornea verticillata group and the control group except the anterior layer in the 0–2-mm (*p* = 0.019) and 2–6-mm (*p* = 0.002) zones. As for the comparison between the no cornea verticillata group and control group, the statistical differences occurred in anterior layer 6–10 mm (*p* = 0.001)—central layer 6–10 mm (*p* < 0.001) total layer (*p* = 0.001) —and posterior layer 2–6 mm (*p* = 0.018), 6–10 mm (*p* < 0.001) and total layer (*p* < 0.001). The comparison among the control group, FD group, and no cornea verticillata group is summarized in Fig. 1.

## Discussions

In this study, we compared the densitometric values between eyes with and without cornea verticillata. The findings suggested that corneal densitometry may be a potential tool for detecting small corneal alterations in patients with FD.

Pathogenic variants cause FD in the gene of alpha-galactosidase A (alpha-Gal A; galactosidase alpha [*GLA*]) mapped to the long arm of the X chromosome (Xq22.1 region) [18]. FD patients may present with a spectrum of clinical manifestations, ranging from the severe classic phenotype in males to asymptomatic disease in some females. However, cornea verticillata is a characteristic sign that appears relatively early in most hemizygous males and a large number of heterozygous females [11, 12]. Furthermore, cornea verticillata rarely occurs in individuals without FD, except for patients using specific drugs, such as amiodarone and chloroquine. Therefore, the representation of cornea verticillata in patient not taking amiodarone or chloroquine is highly sensitive and specific for the diagnosis of FD [12].

Furthermore, early diagnosis of FD is crucial since therapy can be started before severe irreversible organ damage occurred, and thus, it reduces the risk of progression to organ failure [19, 20]. Although it is commonly believed that FD does not affect visual acuity [9, 21], which is consistent with the findings in our study, cornea verticillata changes corneal densitometry. Corneal densitometry gives an indication of the transparency of the cornea. Corneal abnormalities often reflect visible lights and reduce the light scatter of surrounding healthy cornea [22]. In this study, the no cornea verticillata group is statistically different from the control group. This result implies corneal densitometry might discover subclinical cornea verticillata even earlier than the slit-lamp examination. Additionally, the slit-lamp examination has limited diagnostic power in the detection of epithelial deposits in patients with FD. The slit-lamp examination suffers from a high number of false-negative results and consequently a low negative predictive value [23]. The corneal densitometer is a new indicator that has been involved in the diagnosis and evaluation of cornea-related diseases and treatment results. [24–26]. Therefore, corneal densitometry may be performed as a more sensitive tool than a slit-lamp examination to detect epithelial deposits in patients with FD.

However, to date, no studies have demonstrated the connections between corneal densitometric values and FD. In this study, we divided patients into two groups according to the results of the slit-lamp examination, i.e., a no cornea verticillata group and a cornea verticillata group. Furthermore, three zones of the cornea at different depth levels were selected for densitometry, involving the zones with diameter values of 2 mm, 2–6 mm, and 6–10 mm. The 10–12-mm limbal region was deselected since the repeatability in this region is the lowest for Pentacam [27, 28], which may be caused by the examination environment and patient eye movement. A previous study enrolled 445 healthy participants and performed corneal densitometry over the entire 12-mm diameter area of cornea [27]. The densitometric values in the 40- to 50-year-old group were 25.6 ± 3.69 GSU in the anterior layer, 17.38 ± 2.42 GSU in the center layer, and 15.5 ± 2.11 GSU in the posterior layer. In this study, the findings showed that eyes with FD had higher corneal densitometric values than healthy ones mentioned above. In comparison with the control group, the same result appeared except for the central layer. However, in the sub-analysis, there were no differences between the cornea verticillata group and the control group except anterior layer in the 0–2-mm and 2–6-mm zones. This result is most likely due to a small sample, but it meets the pathological characteristics of cornea verticillata.

Histologically, cornea verticillata is located in the epithelium and anterior stroma of the cornea [17], corresponding to the anterior layer and central layer in the Pentacam examination. In this study, significant differences were found between patients with and without cornea verticillata in the anterior layer of the 6–10-mm zone, posterior layer of 6–10-mm zone and total zone, and the central layer of all zones. The results of the central layer in all zones as well as the posterior layer in 0–2-mm and 2–6-mm zones conform to previous histological studies. On the contrary, no significant difference was found in other layers of the anterior layer. This may be due to the micro-pathological changes in FD patients and the false-negative results of slit-lamp examination. We also found the initial diagnosis time was negatively related to the corneal densitometry value in the 0~2-mm and 2~6-mm posterior layer. Because one of our patients has a very early first diagnosis and a small sample size, when abnormal values appear, it is easy to affect our statistical results. When we removed this patient, there was no relationship between the first diagnosis time and corneal optical density. In summary, we assume that the initial diagnosis time was negatively related to the corneal densitometry value in the posterior layer is clinically irrelevant.

This study also has its limitations. Due to the scarceness of FD patients, the sample size of this study is small and may have an impact on the results. Due to the limitation of the sample size and corneal densitometry false-positive results attributed by age, gender, or other relative diseases, further prospective multi-center comprehensive cohort studies that include healthy volunteers and patients with different refractive errors are needed to confirm our current findings. Besides, patients with FD may visit a physician rather than an ophthalmologist, which causes the loss of initial data for ophthalmologists prior to initiation of therapy. Regrettably, we did not find any study on ocular symptoms changes during ERT. Therefore, the cooperation needs to be closer between doctors from different related departments.

In conclusion, FD patients show higher corneal densitometry than healthy people. Corneal densitometry, especially in the anterior and posterior layer, may be a potential tool for early diagnosis of FD in the future and may play a reference role in reminding the progress of FD.

## Data Availability

All data during the study are available from the corresponding author by request.
